# Proteomes of aging and omega-3 supplementation in rat soleus skeletal muscle

**DOI:** 10.1371/journal.pone.0323602

**Published:** 2025-05-27

**Authors:** Francesco Maria Del Re, David W. Russ, Kalina P. Dimova, Stylianos P. Scordilis

**Affiliations:** 1 Department of Biological Sciences, Smith College, Northampton, Massachusetts, United States of America; 2 School of Physical Therapy and Rehabilitation Sciences, University of South Florida, Morsani College of Medicine, Tampa, Florida, United States of America; 3 Ohio Musculoskeletal and Neurological Institute (OMNI), Heritage College of Osteopathic Medicine, Athens, Ohio, United States of America; 4 Program in Biochemistry, Smith College, Northampton, Massachusetts, United States of America; 5 Center for Proteomics, Smith College, Northampton, Massachusetts, United States of America; McMaster University, CANADA

## Abstract

Aging is a pan-organ process with an intricate and multimodal nature. Deciphering the aging phenomenon is complex, yet recent attention is analyzing the potential benefits of non-invasive life adjustments to achieve healthy aging. Omega-3 polyunsaturated fatty acids (FA) have emerged as promising nutraceuticals for a plethora of different medical conditions. In the current study we conducted an in-depth, bottom-up, global, shotgun proteomic study (LC-MS/MS) investigating both the effects of aging on skeletal muscle and the potential alterations due to ω-3 FA. Sprague Dawley rats were fed different diets and divided into four groups (n = 5 per group): adult controls (7–8 months, ADCTL); aged controls (22 months, AGCTL); and adult (ADω3) and aged (AGω3) rats fed an ω-3 supplemented diet. Among the identified 30,000 soleus proteins, our proteomic analysis identified 149 proteins differentially expressed in aging; 207 proteins with aging, but fed ω-3 FA; and 105 and 26 proteins, respectively, when aged and adult rats were fed ω-3 FA. Aging alone (ADCTL/AGCTL) affects many processes: carbohydrate and lipid metabolisms, proteostasis, mRNA processing and sarcomeric proteins. With FA supplementation and aging (ADω3/AGω3) similar processes were affected, but increased chromatin-related protein abundances (methylation or histone deacetylation) were observed in AGω3; while proteins involved in OXPHOS and mitochondrial homeostasis, including mTOR, were more represented in ADω3 rats. Supplementation with FA had a greater effect in aged rats (AGCTL/AGω3) than in adult ones (ADCTL/ADω3). In the ADCTL/ADω3 comparison, modest changes were seen, whereas in the AGCTL/AGω3 comparisons DNA damage repair increased and protein synthesis and degradation were observed. Further, a potential link to enhancement of myogenesis is also evident. The data presented in this work suggest potential beneficial and protective effects of ω3 FA supplementation in the soleus muscle, as well as some potential molecular mechanisms of action.

## Introduction

Aging is an incompletely deciphered, universal and pan-organ process*.* As numerous mechanisms underpinning the etiology of the processes have been certainly unveiled, the multifactorial makeup of the aging phenomenon makes its study complex. Hence, there is a scarcity of treatments for delaying or preventing aging-related disabilities and a need for novel biomarkers. To date, twelve major molecular “hallmarks of aging” have been identified [1]. Among these, chronic inflammation is considered a pivotal signature of aging, also referred to as “inflamm-aging” [2].

In this complex scenario, omega-3 polyunsaturated fatty acids (ω-3 PUFAs) have emerged as promising nutraceuticals by virtue of their anti-inflammatory activities, being suggested worthwhile for the treatment and prevention of a plethora of conditions including cardiovascular diseases [3]. For example, reduced serum cholesterol levels have been noticed in populations who consume a diet rich in protein and fish [4], with the latter being one of the main sources of ω-3 PUFAs. While some clinical case reports have highlighted the need to consider the potential anticoagulant effects of ω-3 PUFAs when consumed at excessive doses and concomitantly taken with Warfarin [5,6], a retrospective cohort analysis found no effects of fish oil on Warfarin control, bleeding incidences and thromboembolic events [7].

A comprehensive literature explaining the molecular mechanisms of ω-3 PUFAs on adult and aged skeletal muscles is still scarce. This paucity of information can be ascribed to the complex mosaic of molecules present in skeletal muscle tissue which creates difficulties in the identification of principal effectors in the ω-3 PUFAs response. Our lab has already demonstrated that ω-3 PUFAs reduce the sarcopenia-associated loss of proteins in the soluble fraction extracted from the medial gastrocnemius [8]. The present study provides information for better understanding the impact ω-3 PUFAs have on the proteome in rat skeletal soleus muscle *in toto*, analyzing both the soluble (sarcoplasmic) and insoluble (sarcomeric) fractions. Our hypothesis is that ω-3 PUFAs alter the skeletal muscle proteome by targeting specific pathways inferring molecular mechanisms that may ameliorate age-related dysregulation. Notably, our study focuses on understanding the physiological changes in the proteome during aging and the potential reparative effects of ω-3 PUFAs, independently from sarcopenia.

We conducted an in-depth bottom-up global shotgun proteomic study comparing solei skeletal muscles of adult (7–8 month) and aged (22 month) Sprague-Dawley rats during aging with and without an ω-3 PUFAs supplemented diet.

## Materials and methods

### Animals, diet, ethical approval

Male Sprague Dawley rats (Harlan), adult (5–6 months at receipt) and aged (20 months at receipt), were housed in 12 h light-dark cycles at 22°C at Ohio University (Athens, OH). The rats were acclimated for two weeks with a purified diet (American Institute of Nutrition rodent diet AIN-93 M; 12.4% protein; 68.4% carbohydrate; 4.1% fat by weight) and water *ad libitum*. Then, rats were placed either on an 8-week control (CTL) diet (AIN-93 M) or an 8-week control diet supplemented with fish oil (FO), with 28.4% eicosapentaenoic acid (EPA) and 12.7% docosahexaenoic acid (DHA) (total 4% fat). This provided a FO dose of ~ 1.22 g/kg bodyweight (bw)/day or EPA dose of 0.35 g/kg bw/day. Our choice of the high FO dose was also based on a study on humans, conducted on cachectic pancreatic cancer patients, which showed that a 3-week intake of 2.2 g of EPA and 0.96 g of DHA per day was well tolerated and instrumental for metabolic normalization [9]. The rats were assigned to four test conditions (n = 5 for each): ADCTL (adult control); AGCTL (aged control); ADω3 (adult rats supplemented with FO); and AGω3 (aged rats supplemented with FO). After 8 weeks, the animals were subjected to injury of the medial gastrocnemius muscle of one of the hindlimbs for another study [10]. Soleus tissue for the current study came from the uninjured hindlimb. Animals were anesthetized (Ketamine/Xylazine 40:10 mg kg^−1^ body mass, intraperitoneally) and euthanized (while still anesthetized) by an intracardiac injection of anesthesia (Euthasol, 100 mg kg^−1^) following tissue collection. All procedures were approved by the Ohio University Institutional Animal Care and Use Committee, and the “Principles of laboratory animal care” (NIH publication No. 86–23, revised 1985).

The principal diagnostic biomarker of sarcopenia, loss of skeletal muscle mass, was not detected in our samples and “sarcopenia index” [11] was reported (S1 Table). The absence of sarcopenia in aged soleus muscle aligns with previous studies [12,13].

### Protein extraction

Fifteen volumes (µL per mg tissue) of extraction buffer (10 mM NaPO_4_, pH 7.0; 2 mM EDTA; 10 mM NaN_3_; 140 mM NaCl; 1% NP-40, 1 X protease and phosphatase inhibitor, Cat. #: 78442, ThermoFisher) were added to the muscle pulp. The extract was homogenized on ice with 3 x 15 second bursts (via Polytron), vortexed every 15 minutes for 1 hr, and then centrifuged for 30 minutes at 4°C at 15,600 rcf. Supernates (sarcoplasmic fractions) were separated from pellets (sarcomeric fractions). Supernate samples were further precipitated by addition of three volumes of cold (-20°C) acetone, vortexed for 10–15 seconds, and centrifuged for 10 seconds at 4°C to form a pellet. Then, the supernate was gently poured off and the pellet was resuspended to the original volume in 18 MΩ H_2_O. The acetone precipitation step was repeated. Sarcoplasmic and sarcomeric fractions were resuspended in a modified Laemmli sample buffer containing a final concentration of 5% SDS [14]. Samples were further homogenized with a motorized pellet pestle and heated at 100°C for 2 minutes.

### Total protein estimation

A modified version of the Lowry protein estimation protocol [15] was used to estimate the protein concentration of both fractions. Prior to the Lowry assay, a protein precipitation in trichloroacetic acid (50%) was performed to remove interfering substances [8]. One mg mL^-1^ crystalline bovine serum albumin (BSA) was used to create a reference standard curve.

### SDS-PAGE

**Fig 1:**
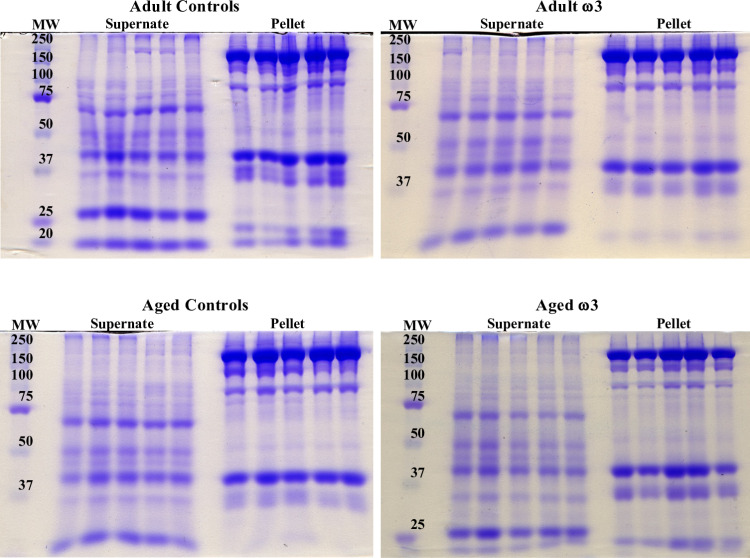
One-dimension SDS-PAGE showing the supernate (sarcoplasmic) and pellet (sarcomeric) fractions. Each gel shows one condition (group) with five biological replicates per fraction. Each lane contains 20 μg of proteins. This experiment validates our separation method showing the partition of sarcoplasmic and sarcomeric proteins.

The SDS-PAGE technique [14] was used to validate the correct separation of the extract proteins in the supernate and pellet fractions. Each lane of the gel (5% stacking and 10% separating) was loaded with 20 µg of proteins, as well as with a molecular weight standard in the first lane (5 µL). The gels were stained (45.5% methanol, 45.5% 18 MΩ H_2_O, 9.0% acetic acid, 0.25% Coomassie Brilliant Blue R250) overnight and then destained twice (45.5% methanol, 45.5% 18 MΩ H_2_O, 9.0% acetic acid), replacing the destain at 3 hr. A subsequent destaining (5% methanol, 90% 18 MΩ H_2_O, 5% acetic acid) occurred overnight. Next, the gel was stored in 7.5% acetic acid for one day prior to being scanned (Fig 1).

As expected, contractile proteins such as myosin and actin (Mr ≅ 200 kDa and 43 kDa, respectively) were most abundant in the pellet fraction. This trend was consistent for all the samples. Most notably, myosin was almost exclusively present in the pellet fractions; whereas actin was found in both supernate and pellet, but still predominantly found in the latter. This ubiquitous presence of actin can be explained by its dual role as a pivotal component of both the muscle cytoskeleton and sarcomere, as well as sarcoplasmic monomeric actin.

### S-TRAP and tryptic digestion

Two-hundred µg of total extracted proteins from each sample were reduced by adding fresh DL-1,4-dithiothreitol to a final concentration of 20 mM. Samples were vortexed and heated at 95°C at 300 rpm for 9 minutes and left to equilibrate to room temperature (RT). Next, samples were alkylated with iodoacetamide (40 mM), followed by vortexing and a 30 min incubation in the dark. Samples were then processed according to the manufacturer’s protocol “S-TRAP mini (100 µg - 300 µg) digestion protocol” (Protifi), with the exception that the amount of trypsin was scaled down to 4 µg (1:50 wt:wt). After being lyophilized and concentrated *in vacuo*, the obtained peptides were resuspended in 150 µL of 100 mM triethylammonium bicarbonate.

### Total peptide quantification

A Quantitative Fluorometric Peptide Assay (Cat #: 23290, Thermo Scientific) was used to analyze the concentration of the tryptic peptides. Fluorescence was measured using Ex/Em at 390 nm/475 nm via a Spectramax MS Multi-Detection Microplate Reader (Software SoftMaxPro 7.0).

### Tandem mass tag (TMT) labeling

Lyophilized peptides from the sarcoplasmic and sarcomeric fractions were resuspended in 100 mM triethylammonium bicarbonate. A 0.8 mg/tube TMT10plex label reagent set (Cat #: 90110; Lot #: UI281756, Thermo Scientific) was reconstituted in 41 µL of anhydrous acetonitrile. In line with previous TMT experiments [16,17], 10 µg of peptides were labelled with 4.1 µL of TMT tags. The mix was incubated for 1 hr and the reaction was quenched by the addition of 2 µL of 5% hydroxylamine for 15 minutes at RT. Samples were then combined according to the experimental comparisons and 8 final master mixes were obtained. The master mixes were concentrated and lyophilized *in vacuo* and stored at 4°C.

### High pH reversed-phase peptide fractionation

A high pH reversed-phase peptide fractionation kit (Cat. #: 84868, Thermo Scientific) was used to separate peptides based on their hydrophobicity to increase the resolution of the peptide identification. One hundred µg of each of the lyophilized master mixes were dissolved in 300 µL of 0.1% trifluoroacetic acid and the manufacturer’s protocol was followed. The fractions were lyophilized *in vacuo* and stored at -20°C.

### LC-MS/MS analysis, protein annotation, and quantification

Each lyophilized peptide comparison mixture was dissolved in 10 µL of 0.1% formic acid and analyzed on a Q Exactive HF-X Orbitrap Mass Spectrometer coupled with a Thermo Scientific Easy-nLC 1000. Next, 2 μL of each fraction were desalted online on a nanoViper Acclaim PepMap100 C18 column (100 μm i.d. x 2 cm) and separated on a nanoViper Acclaim PepMap RSLC C18 column (75 μm i.d. x 50 cm) at a flow rate of 300 nL/min. A two-step 120 min gradient was used: 5–22% of 0.1% formic acid in acetonitrile for 0–100 min and 22–35% of 0.1% formic acid in acetonitrile for 100–120 min (mobile phase A: 0.1% formic acid in water). Samples were injected into the mass spectrometer through a Nanospray Flex ion source with a stainless-steel emitter (O.D. 150 μm, i.d. 30 μm) at a spray voltage of 1.9 kV.

The raw files were then analyzed by Proteome Discoverer 2.4.0.305 (Thermo Scientific). The TMT 10plex_UI281756 quantitation channels were: 126, 127N, 127C, 128N, 128C, 129N, 129C, 130N, 130C, 131 (with impurity correction factors accounted). Groups were sorted by age and diet depending on the comparisons, and the protein annotation was conducted by the Sequest HT database against the *Rattus norvegicus* database (NcbiAV TaxID = 10116, v. 2017-10-30) and Percolator validation (validation based on q-value, concatenated target/decoy selection, maximum ΔCn 0.05). The confidence thresholds used were strict and relaxed FDRs (0.01 and 0.05, respectively). Analysis of variance (ANOVA, individual proteins) was chosen as method for hypothesis testing. Settings such as trypsin, precursor mass tolerance 20 ppm, fragment mass tolerance 0.5 Da and modifications were applied to the search. Among the latter, static and dynamic modifications were included (static: TMT6plex, + 229.163 Da (any N-terminus); TMT6plex, + 229.163 Da (K); carbamidomethyl, + 57.021 Da (C); dynamic: oxidation, + 15.995 Da (M); deamidated, + 0.984 Da (N, Q); acetyl, + 42.011 Da (N-Terminus); met-loss, -131.040 Da (M); met-loss+acetyl, -89.030 Da (M)). Relative to the reporter quantification, the following thresholds (%) were used: 30 (co-isolation), 10 average reporter S/N, and 65 for SPS mass matches.

Results were sorted for unique high confidence peptide and statistical significance was assigned as +/- 1.5-fold change (FC) and -log_10_ p-value (p) ≤ 0.05. Protein-protein interactions (physical and functional) networks were illustrated via String database 12.0 [18] setting the following parameters (organism: *Rattus norvegicus*; minimum required interaction score: high confidence 0.700; false discovery rate stringency: high, 1%; protein-protein interaction, PPI, enrichment p-values were also reported for each comparison).

The proteins visualized via String database 12.0 were also enriched for biological processes and pathways by combining the information obtained from ShinyGO 0.80 [19] (species *Rattus* *norvegicus* with ENSEMBL as source; the top 20 pathways with a minimum size of 2 were shown; pathways were filtered by FDR cutoff 0.05 and sorted by fold enrichment, FE; GO Biological Process and KEGG pathway databases were interrogated) and a manual search through the published literature.

### Quantitative immunoblotting

After LC-MS/MS analysis, significant differential protein abundance and expression of selected proteins were investigated and validated by quantitative immunoblotting [20]. Specifically, three proteins were tested: NADH (S1 Fig) and histone H3 (S2 Fig) which showed significant relative differential abundances at the LC-MS/MS; and GAPDH which was not both significant and differentially expressed (S3 Fig).

The transfer was carried out at 4°C at 50 V in cold transfer buffer (192 mM glycine, 25 mM Tris, 15% v/v methanol) for 1.5 hr. After the transfer, the gel was stained with Coomassie Brilliant Blue R250 (*vide supra* SDS-PAGE section for details) to assess the success of the protein transfer. The fraction of the total proteome transferred onto the PVDF membrane (S4 Fig) was manifested via MemCode reversible protein stain (Cat. #: 24585, Thermo Scientific).

The primary antibody (Ab) was prepared in TBS-T/BSA (1% BSA) buffer according to the following dilutions (histone H3 1:1,000, Cell Signaling Technology, mAb, Cat. #: 3638; NADH dehydrogenase ubiquinone flavoprotein 2 1:2,500, ProteinTech, polyAb, Cat. #: 15301-1-AP; GAPDH 1:75,000, Millipore, mAb, Cat. #: MAB374). The secondary antibody was prepared in TBS-T/BSA (1% BSA) buffer according to the following dilutions (Cell Signaling, Goat anti-Rabbit, 1:5,000, Cat. #: 7074 for NADH dehydrogenase ubiquinone flavoprotein 2; ProteinTech, Goat anti-Mouse, 1:5,000 and 1:2,500, Cat. #: SA00001-1 for histone H3 and GAPDH respectively). The membrane was developed with TMB Peroxidase (Cat. #: 50-77-00, KPL).

The same membrane was stripped and reprobed with multiple antibodies. Stripping Buffer (Mild Stripping Abcam, 0.1% SDS, 1% Tween 20, 1.5% glycine, pH 2.2) was added *quantum satis* to cover the membrane, which was then incubated in a water bath at 50˚ C for 45 minutes. Next, the membrane was washed with 18 MΩ H_2_O for 1 minute and three times with TBS-T, 5 minutes each. Finally, the membrane was washed once with TBS for 5 minutes and then blocked and reprobed with a new antibody (as described above).

The pixel intensities of each band were integrated via ImageJ software and normalized to the total protein content per lane from the MemCode via JMP 12.2.0. Data were plotted with GraphPad Prism 10.2.3 (experimental design: unpaired; Gaussian distribution: parametric test; unpaired t test with Welch’s correction; Two-tailed *p-*value; p-value style: *p* = 0.1234 not significant ns, *p = 0.0332, **p = 0.0021, ***p = 0.0002, and ****p < 0.0001).

## Results

Each muscle extract was partitioned into two different fractions: a pellet (sarcomeric, containing insoluble cellular contractile components) and a supernate (sarcoplasmic, including soluble/cytoplasmic proteins). This separation aimed at increasing the dynamic range of identification of the least abundant musculoskeletal proteins at LC-MS/MS analysis. Next, the supernate and pellet fractions were analyzed together for the final proteome analysis via a bottom-up, global, shotgun proteomic analysis comparing aging and ω-3 PUFAs diet. Rat samples were divided into four different groups (n = 5 for each group): adult controls (7–8 months, ADCTL); aged controls (22 months, AGCTL); and adult and aged rats fed an ω-3 supplemented diet (ADω3, AGω3).

Differences in relative protein abundances retrieved with LC-MS/MS analysis were investigated. For clarity, each comparison is discussed below separately. For each comparison only proteins identified by unique peptides and with both a 1.5-FC in differential expression between the two groups and a -log_10_ p ≤ 0.05 were further investigated (Table 1). These proteins were then visualized via volcano plots generated through Proteome Discoverer 2.4.0.305.

**Table 1:** Proteins identified via LC-MS/MS for each comparison (supernate and pellet). Of all the total proteins, only those deriving from unique peptides were further analyzed. A criterion of 1.5-FC and p ≤ 0.05 was applied to enrich for proteins within each comparison.

Comparison	Fraction	Total proteins	Unique peptides	Total, up and down regulated proteins
**ADCTL/AGCTL**	Supernate	18,499	3,362	**149** ↑ 34 ↓ 115
	Pellet	11,713	1,802	
**ADω3/AGω3**	Supernate	18,548	3,309	**207** ↑ 43 ↓ 164
	Pellet	11,777	1,828	
**ADCTL/ADω3**	Supernate	16,599	2,855	**26** ↑ 6 ↓ 20
	Pellet	12,554	1,960	
**AGCTL/AGω3**	Supernate	19,003	3,437	**105** ↑ 62 ↓ 43
	Pellet	12,643	2,008	

### Adult control/aged control

**Fig 2:**
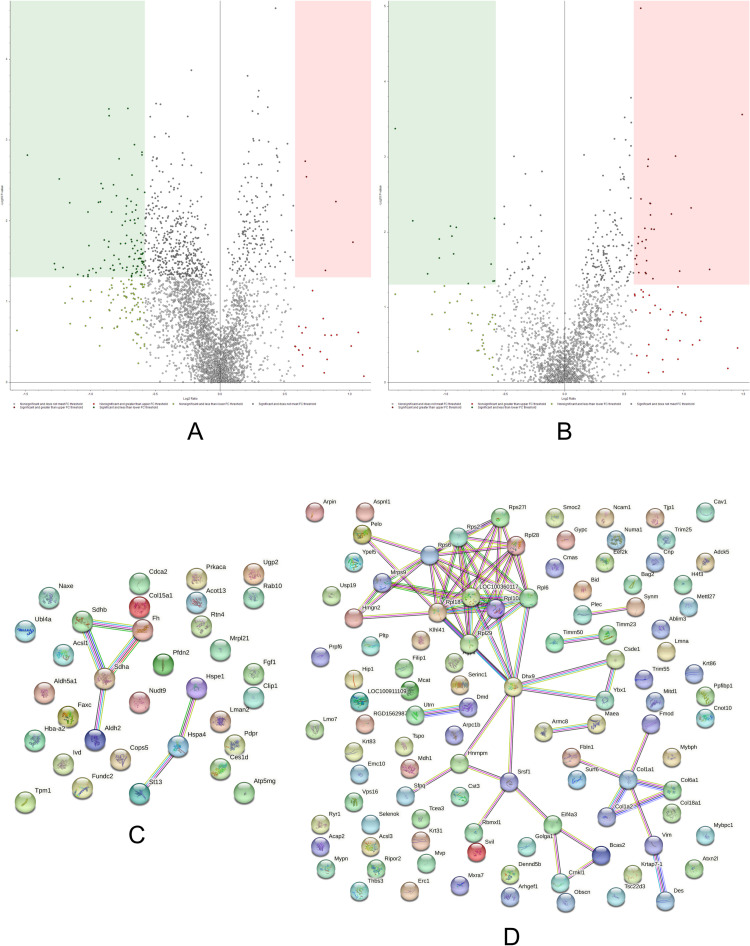
Volcano plots and String networks for ADCTL/AGCTL comparison. **(A, B)** The volcano plots display both supernate (sarcoplasmic, A) and pellet (sarcomeric, B) fractions. The proteins in the colored boxes meet both the significance (-log_10_ p ≤ 0.05, Y axis) and differential abundance (log_2_ Fold Change = 0.58, X axis) criteria. The sarcoplasmic fraction (A) indicated 5 up-regulated proteins (175 ≤ p, 26 ≥ FC) and 101 down-regulated proteins (514 ≤ p, 185 ≥ -FC). The sarcomeric fraction (B) showed 29 up-regulated proteins (164 ≤ p, 60 ≥ FC) and 14 down-regulated proteins (46 ≤ p, 50 ≥ -FC). **(C, D) String network (String 12.0) of the significant, up (C) and down (D) regulated proteins in ADCTL/AGCTL.** The PPI enrichment p-value for C was 0.126; for D was 3.73e-08.

The ADCTL *versus* AGCTL comparison aims to evaluate proteome changes as a result of aging. According to our TMT data (S1 and S2 Files), the effects of aging on rat solei muscles are strong, with a plethora of proteins showing differential abundances (Fig 2).

A limited number of proteins were found up-regulated in the ADCTL groups, and among these several enzymes belonging to the citric acid cycle (CAC, enrichment FDR 8.1E-04, FE 82.4) and other metabolic pathways stand out. Interestingly, most of these enzymes show nearly identical abundance ratios such as succinate dehydrogenase [ubiquinone] iron-sulfur subunit, mitochondrial precursor (1.648), fumarate hydratase, mitochondrial (1.648) and succinate dehydrogenase [ubiquinone] flavoprotein subunit, mitochondrial precursor (1.52). Consistent with the increased expression of these metabolic enzymes, adult rats also show a greater abundance of mitochondrial pyruvate dehydrogenase phosphatase regulatory subunit (1.656), which is pivotal for regulating the CAC. This enzyme controls and activates via post-translational modification (PTM) the multi-enzyme pyruvate dehydrogenase, which fuels the CAC providing the substrate acetyl CoA.

Furthermore, a link between OXPHOS and lipid metabolism emerges from the data. In fact, carboxylesterase-like precursor (1.757) and long-chain-fatty-acid-CoA ligase 1 (1.54, also known as acyl-CoA synthetase 1, ACSL1) were both found increased in adult rats. Acyl-CoA synthetase first activates a fatty acid producing a fatty acyl-CoA, which can be directed towards lipid catabolism (fatty acid degradation, enrichment FDR 3.5E-02, FE 33.2). In turn, the acetyl-CoA produced via beta-oxidation can be catabolized replenishing the CAC and the electron transport chain. However, acyl-coenzyme A thioesterase 13 (1.534), which mediates the opposite reaction of deactivation of acyl-CoAs [21], was also increased. This could reflect the fine level of regulation of these processes. The sustained oxidative metabolism rate of adult rat soleus skeletal muscle might allow lipid synthesis without experiencing lipotoxicity, which bespoke mechanism may be the basis for the athlete’s paradox [22].

The main repercussion of relying on OXPHOS as primary energy source is the generation of reactive oxygen species within the electron transport chain. Aldehyde dehydrogenase isoenzymes can act as detoxifying mechanisms [23]. Adult rats show an up-regulation of aldehyde dehydrogenase, mitochondrial isoform X1 (1.537, predicted), which could be cytoprotective against oxidative stress. Along with detoxifying enzymes, mitochondrial proteostasis can be maintained by additional parallel strategies. Several chaperones involved in protein folding (enrichment FDR 1.3E-02, FE 16.3) were found increased in adult rats such as the 10 kDa heat shock protein, mitochondrial (1.595) and heat shock 70 kDa protein 4 (1.557). Other chaperones and co-chaperones found up-regulated were Hsc70-interacting protein (1.683), and prefoldin subunit 2 (1.606).

Aged rats do not rely on carbohydrate metabolism to the same extent as adult rats. This metabolic behavior might be due to the aged-related mitochondrial dysfunction, which ultimately leads to a decreased OXPHOS activity [24]. Our observation also agrees with another study which investigated the aging process in soleus muscle with TMT labeling [25].

In these data, markers of aging are found in the accumulation of many structural molecules such as desmin, vimentin, plectin isoform X1 (intermediate filament organization pathway, enrichment FDR 7.1E-05, FE 25.7). Desmin (0.448), an intermediate filament protein, localizes at the Z-lines of skeletal muscles and it is involved in muscle contraction connecting adjacent myofibrils together, and also playing a role in efficient excitation-contraction coupling [26,27]. The increased presence of desmin abundance could be explained by typical phenomena of aging such as loss of myofilaments [28] and/or denervation-reinnervation processes [29]. At the sarcomeric level, myosin-binding protein H isoform X1 (0.482, predicted); myosin-binding protein C, slow-type isoform X6 (0.519, predicted); myopalladin isoform X1 (0.566, predicted) were also found up-regulated in aged rats. Despite the fact the soleus in Sprague-Dawley rats is more than 80% type I fibers [30], we still report evidence of fiber changes with aging with an increased abundance of the slow-type myosin-binding protein C. Interestingly, dynamic processes involving the cytoskeletal actin seem to take place in aged rats. The fine-tuned regulation of these actin processes can be appreciated by the increased presence of both activator and inhibitor molecules such as Arp 2/3 complex subunit 1B (0.566) and its inhibitor arpin (0.663); the small GTPase Rho protein activator Rho guanine nucleotide exchange factor 1 (0.538) and Rho inhibitor RHO family-interacting cell polarization regulator 2 (0.611).

Another important hub of proteins found to increase during aging are the mRNA processing (spliceosome, enrichment FDR 6.5E-04, FE 10.9) and protein synthesis processes (ribosome, enrichment FDR 5.1E-06, FE 12.3). Finally, at the extracellular level, many different collagen proteins were found up-regulated in the aged group suggesting a reorganization of the malleable and variegated extracellular matrix (ECM organization, enrichment FDR 6.7E-04, FE 7.3).

### Adult ω-3/aged ω-3

**Fig 3:**
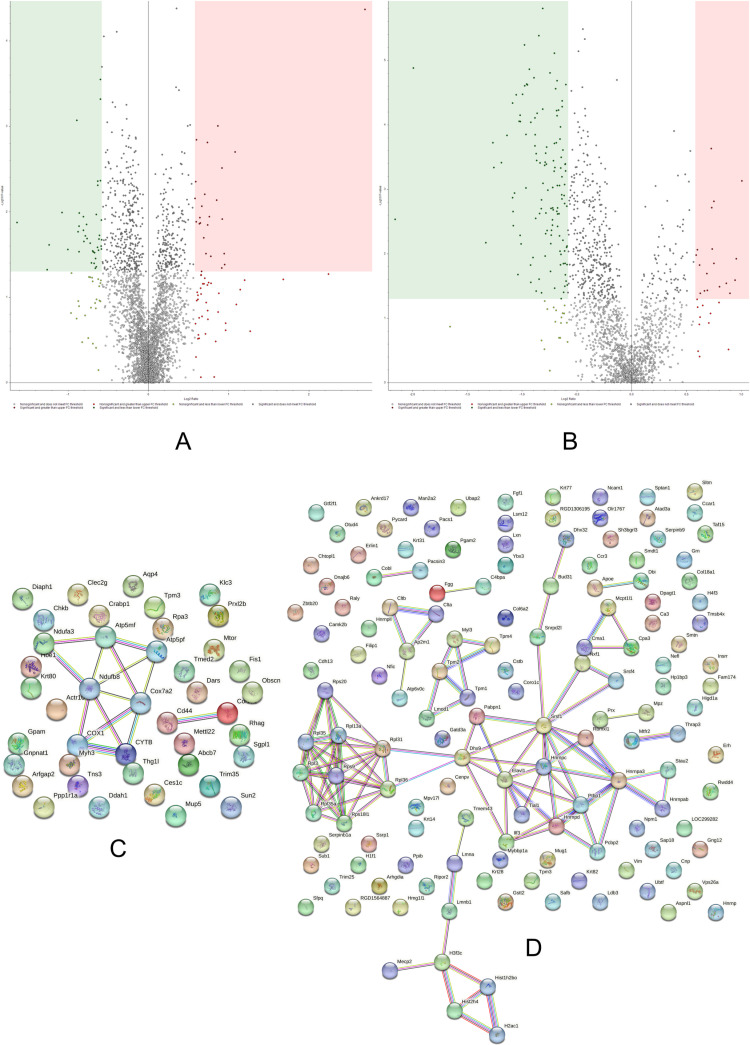
Volcano plots and String networks for ADω3/AGω3 comparison. **(A, B)** The volcano plots display both supernate (sarcoplasmic, A) and pellet (sarcomeric, B) fractions. The sarcoplasmic (A) fraction indicated 25 up-regulated proteins (236 ≤ p, 63 ≥ FC) and 39 down-regulated proteins (362 ≤ p, 64 ≥ -FC). The sarcomeric (B) fraction showed 18 up-regulated proteins (97 ≤ p, 29 ≥ FC) and 125 down-regulated proteins (491 ≤ p, 143 ≥ -FC). **(C, D) String network of the significant, up (C) and down (D) regulated proteins in ADω3/AGω3.** The PPI enrichment p-value was 2.77e-06 for C and <1.0e-16 for **D.**

The ADω3 *versus* AGω3 comparison aims to evaluate the possible muscular-protective effects of ω-3 PUFAs supplementation on the proteome of rat solei skeletal muscles during aging. The TMT data (Fig 3; S3 and S4 Files) suggest different responses and utilization of fish-oil supplementation by adult and aged rats.

Adult and aged rats elicit a plethora of different responses to the ω-3 PUFAs supplementation. In this complex scenario, adult rats fed a ω-3 PUFAs rich diet increase energy metabolism through OXPHOS (enrichment FDR 8.9E-03, FE 15.7). Several enzymes of the respiratory pathway were found up-regulated with similar values such as NADH dehydrogenase [ubiquinone] 1 beta subcomplex subunit 8, mitochondrial (1.665), NADH dehydrogenase [ubiquinone] 1 alpha subcomplex subunit 3 (1.683, predicted) and cytochrome c oxidase subunit 7A2, mitochondrial precursor (1.683).

**Fig 4:**
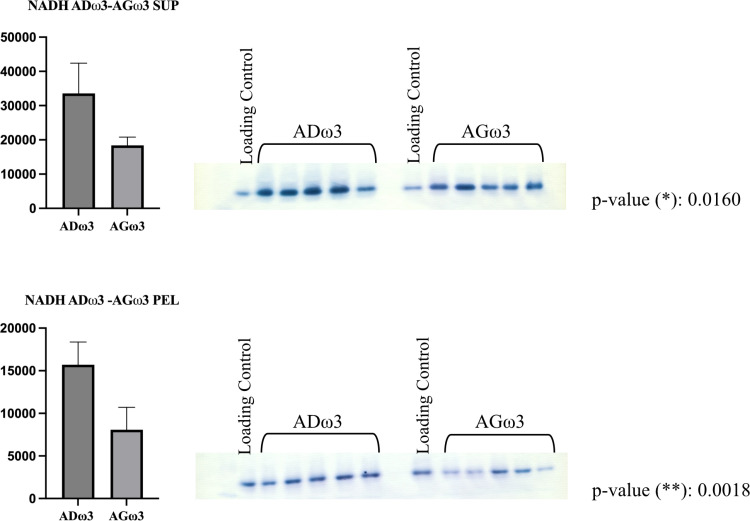
Immunoblots detecting the expression of NADH dehydrogenase ubiquinone flavoprotein 2. Means with standard deviations (SD) are reported. Groups and arbitrary units (pixel intensities) are reported on the x and y axes, respectively.

Relative to NADH, the protein differential expressions quantified by immunoblots (Fig 4) meshed with the trends of differential relative abundances detected by the LC-MS/MS experiment. Thus, the data suggest that ADω3 rats have a more sustained expression of NADH dehydrogenase ubiquinone flavoprotein 2 with respect to their counterpart AGω3.

Parallelly, another interesting enzyme found with a > 6-fold presence in ADω3 compared to the aged counterparts is glycerol-3-phosphate acyltransferase 1, mitochondrial (GPAT1, 6.495). GPAT1 regulates the first rate-limiting reaction of the *de novo* synthesis pathway of glycerolipids (lysophosphatidic acids, LPAs) via esterification of the β-oxidation’s byproduct glycerol-3-phosphate with a fatty acyl-CoA. Another interesting link emerging from the literature is the involvement of GPAT activity in mitochondrial morphology and fusion [31]. The latter observation leads to fascinating scenarios relative to mitochondrial remodeling and dynamics occurring after diet supplementation. According to our data, adult rats fed a ω-3 diet show an increased presence of mitochondrial fission 1 protein isoform X1 (1.82, predicted) and serine/threonine-protein kinase mTOR (1.864). The mechanistic/mammalian target of rapamycin (mTOR) pathway has been demonstrated to stimulate mitochondrial fission [32]. Furthermore, probable tRNA (His) guanylyltransferase (1.671) was also found increased, which protein seems to be involved in mitochondrial fusion [33]. This dynamic remodeling of mitochondria via fission and fusion could be explained as an attempt of adult skeletal muscle cells to adequately adapt to changes in respiration after ω3-PUFAs supplementation.

**Fig 5:**
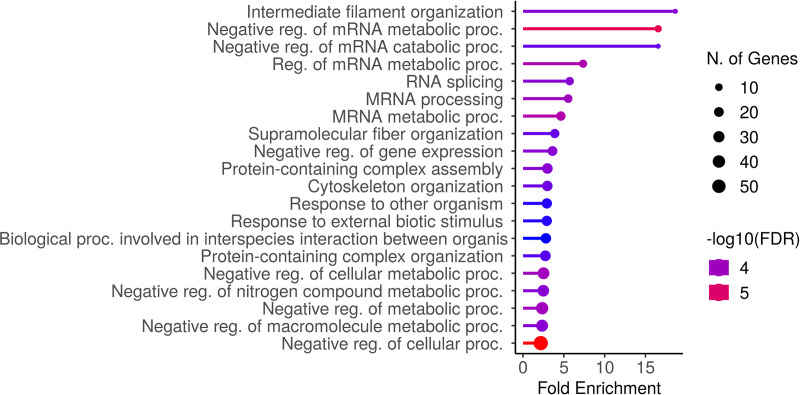
Barplot showing the enriched and ranked pathways in AGω3 rats. After proteomic analysis, proteins were enriched via ShinyGO 0.80 using GO Biological Process pathway database. Pathways shown in the enrichment-barplot were selected by FDR and sorted by fold enrichment (figure generated via ShinyGO 0.80).

Relative to the adult counterparts, aged rats respond differently to nutritional perturbations due to ω-3 PUFAs. Indeed, AGω3 rats show several up-regulated proteins classifiable under three main processes: mRNA processing (enrichment FDR 1.0E-04, FE 5.5) and negative regulation of mRNA metabolic processes (enrichment FDR 5.2E-06, FE 16.6), negative regulation of gene expression (enrichment FDR 1.7E-04, FE 3.6) and intermediate filament organization (enrichment FDR, 1.7E-04, FE 18.7) (Fig 5).

**Fig 6:**
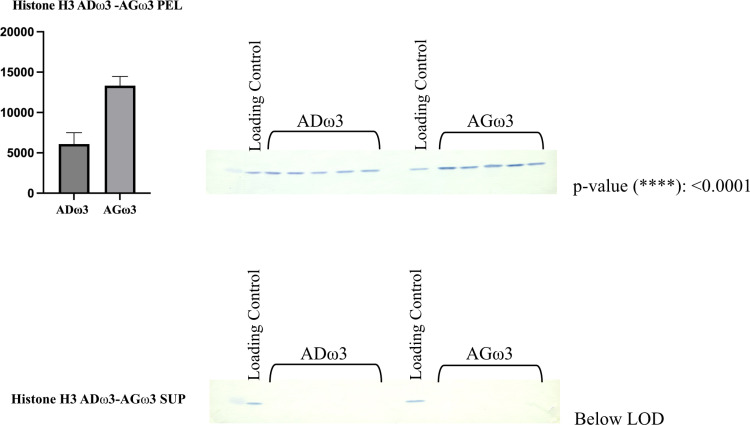
Immunoblots detecting the expression of histone H3. Means with standard deviations (SD) are reported. Groups and arbitrary units (pixel intensities) are reported on the x and y axes, respectively. As expected, no bands were detected in the supernates for the comparison ADω3-AGω3.

Transcription and mRNA processing are also accompanied by a pronounced increased expression of ribosomal and nucleosomal proteins in AGω3 rats such as, to mention some, 60S ribosomal protein L35 (0.418), histone H2A type 4 (0.471), histone H3.3-like (0.508, predicted), chromatin target of PRMT1 protein isoform X1 (0.517, predicted), histone H1.0 (0.539). We decided to test histone H3 via immunoblot and confirmed the trend of differential expression attributed by LC-MS/MS quantitation analyses (Fig 6). As expected, Histone H3 was only detected in the pellet fractions. These results further corroborate the correct protein separation between the sarcomeric (pellet) and sarcoplasmic (supernate) fractions

These findings could also possibly link ω-3 PUFAs to nutri-epigenomics [34], which inferral is further suggested by the increased abundance in AGω3 rats of histone deacetylase complex subunit SAP18 (0.529) and methyl-CpG-binding protein 2 (0.57). Unfortunately, little is known in the literature regarding the contribution of ω-3 PUFAs in controlling and modulating skeletal muscle cell epigenomics. *Cum multis aliis* studies were mainly carried out in cancer models with the purpose of finding a potential antitumorigenic and chemoprotective role of fish oil in coadministration with different nutrients [35].

The ability of ω-3 PUFAs to potentially control chromatin remodeling could ultimately yield to a better DNA accessibility. Facilitates chromatin transcription (FACT) complex subunit structure specific recognition protein 1 (SSRP1) (0.606) was found increased in the AGω3 group relative to the ADω3 one. FACT consists of two heterodimeric subunits SSRP1 and suppressor of Ty 16 (SPT16) and promotes “productive transcription” [36] by modulating the chromatin structure during the transcription of specific genes. The expression of FACT is tightly controlled, and it is usually limited to early stages followed by a decline during differentiation and senescence [37,38]. Strikingly, in our data FACT is higher in AGω3 rats than ADω3, leading to speculation about ω3-PUFAs as potential anti-aging compounds.

Besides FACT, transcript elongation is also supported by numerous other proteins which were found increased in expression in AGω3 compared to ADω3. More specifically, these proteins are associated with mRNA processing and transcription. Of note, Elav-like protein 1 (0.551) is pivotal for myogenesis and differentiation, being found increased in cytoplasmic expression early after damage in regenerating myofibers [39]. The potential scenario of ω-3 PUFAs affecting muscle regeneration during aging can be inferred.

With all these processes being up-regulated, our data also show that AGω3 rats contain an abundance of glutathione S-transferase theta-2 (GSTT2, 0.565). This antioxidant enzyme is effective in detoxifying and preserving DNA integrity by reducing genotoxic insults [40]. In this context, GSTT2 could be instrumental for allowing all these processes to occur without accumulating considerable DNA damage. Another possible anti-DNA damage defensive mechanism in AGω3 is in the increased expression of high mobility group B1 protein (0.528), which protein seems to show a protective effect against DNA insults [41].

Finally, proteins belonging to sarcomere organization and muscle contraction were also increased in AGω3 rats. These proteins include myosin light chain 3 (0.664) and dynamic actin binding proteins such as tropomyosin beta chain isoform Tpm2.2st (0.58); tropomyosin alpha-4 chain (0644); tropomyosin alpha-1 chain isoform Tpm1.3sm (0.607); LIM domain-binding protein 3 isoform 4 (0.5); leiomodin-1 isoform X1 (0.545, predicted); and coronin-1C (0.397). Conversely, in ADω3 rats tropomyosin alpha-3 chain isoform X8 (1.616, predicted) was increased.

### Adult control/adult ω-3

**Fig 7:**
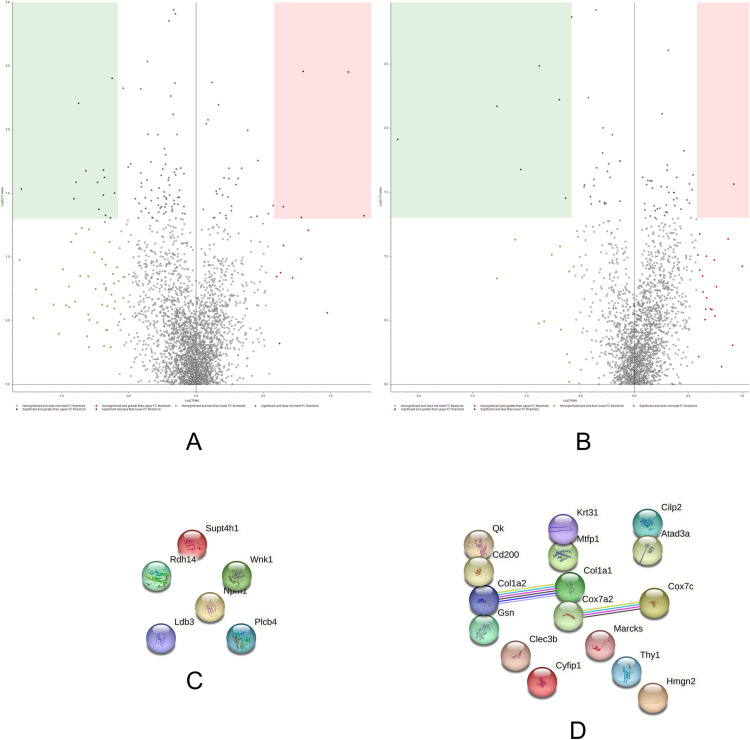
Volcano plots and String networks for ADCTL/ADω3 comparison. **(A, B)** The volcano plots display both supernate (sarcoplasmic, A) and pellet (sarcomeric, B) fractions. The sarcoplasmic (A) fraction indicated 5 up-regulated proteins (34 ≤ p, 13 ≥ FC) and 14 down-regulated proteins (85 ≤ p, 56 ≥ -FC). The sarcomeric (B) fraction showed 1 up-regulated protein (39 ≤ p, 20 ≥ FC) and 6 down-regulated proteins (29 ≤ p, 17 ≥ -FC). **(C,D) String network of the significant, up (C) and down (D) regulated proteins in ADCTL/ADω3.** No interactions were found in the ADCTL basal group if compared to ADω3, showing a PPI enrichment p-value of 1 for **C.** The PPI enrichment p-value for D was 0.0496.

As expected, the TMT data (S5 and S6 Files) suggest that ω-3 PUFAs induced modest changes in adult rats with most of the proteins remaining unaltered when compared to the control group (Fig 7).

The mild effects of ω-3 PUFAs on adult rats could be interpreted as a lesser susceptibility and necessity for the antioxidant and anti-inflammatory properties of ω-3 PUFAs compounds. Hence, adult rats may not exhibit an accumulation of aging-related events so as to demand a significant use of ω-3 PUFAs other than for energy pathways. In keeping with this inference, our data suggest that, after ω-3 PUFAs supplementation, ADω3 rats show increased abundances of different cytochrome isoforms, such as cytochrome c oxidase subunit 7C mitochondrial which showed a greater expression in both pellet (0.219) and supernate (0.628), and cytochrome c oxidase subunit 7A2, mitochondrial precursor (0.618).

In parallel with the accrued respiratory phenotype, changes in mitochondrial homeostasis and remodeling occur as well. In this context, mitochondrial fission process protein 1 (0.567) and ATPase family AAA domain-containing protein 3 (0.607, also referred to as ATAD3) decreased in abundance in the ADCTL/ADω3 ratio. Notably, ATAD3 engages different partners within mitochondria [42], and could participate to mitochondrial shaping [43].

Another important category of proteins showing a consistent increased expression in ADω3 rats compared to the control group is represented by the ECM structural proteins. The increased presence of collagen after ω-3 PUFAs supplementation might suggest a role of ω-3 PUFAs in conferring different physio-chemical properties to the ECM. The consequent remodeling could be instrumental for structuring an extracellular matrix more prone to resist different contractile stresses. Evidence shows external stimuli such as exercise are able to promote ECM adaptation and remodeling [44]. Furthermore, the extracellular protein tetranectin (0.65, predicted) increased in ADω3, and it is involved in myogenesis and muscle regeneration by localizing at the musculo-tendinous and developing myofascial junctions [45]. Interestingly, adult ω-3 rats also show a significant lower abundance of nucleophosmin-like isoform X2 (2.379 in the ADCTL/ADω3 comparison, predicted). Reduced levels of nucleophosmin have been found to be a mechanism for inducing myogenesis in C2C12 cell line and formation of muscle cells [46].

### Aged control/aged ω-3

**Fig 8:**
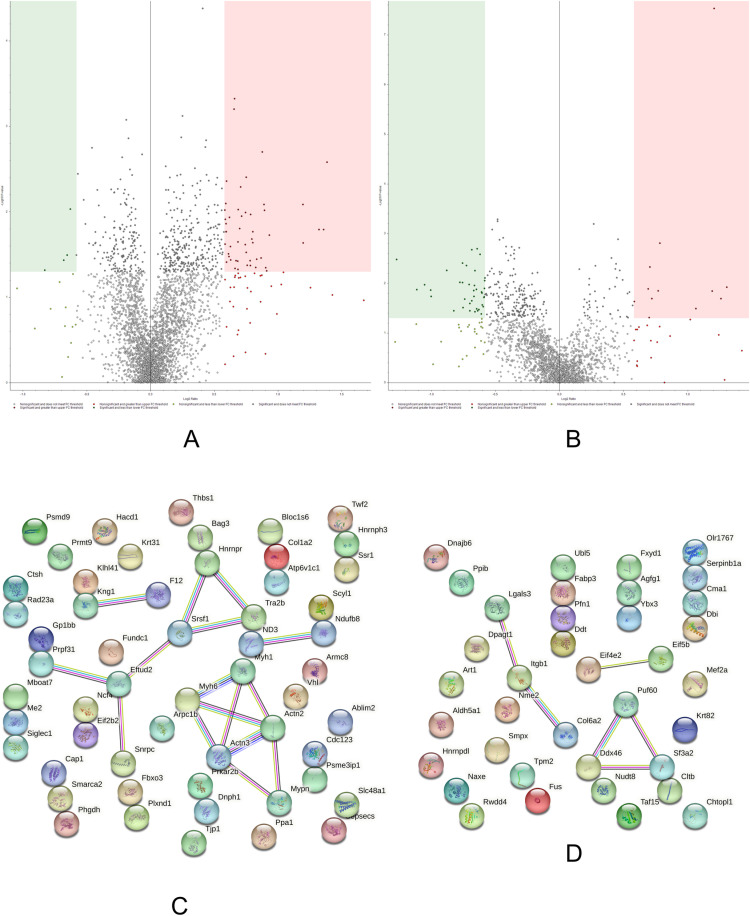
Volcano plots and String networks for AGCTL/AGω3 comparison. **(A,B)** The volcano plots display both supernate (sarcoplasmic, A) and pellet (sarcomeric, B) fractions. The sarcoplasmic fraction indicated 50 up-regulated proteins (338 ≤ p, 83 ≥ FC) and 4 down-regulated proteins (167 ≤ p, 16 ≥ -FC). The sarcomeric fraction showed 12 up-regulated proteins (53 ≤ p, 30 ≥ FC) and 39 down-regulated proteins (221 ≤ p, 63 ≥ -FC). **(C,D) String network of the significant up-regulated (C) and down-regulated (D) proteins in AGCTL/AGω3.** The PPI enrichment p-value was 1.85e-05 for C and 0.00233 for **D.**

The AGCTL *versus* AGω3 comparison (Fig 8; S7 and S8 Files) evaluates the effects of ω-3 PUFAs supplementation on the proteome of aged rat solei skeletal muscles.

Ω-3 PUFAs seem to be beneficial during aging. For example, ω-3 PUFAs fed aged rats do not rely on DNA repair and protein turnover mechanisms to the same extent as the AGCTL group. Furthermore, our data suggest a potential association between ω-3 PUFAs and adult skeletal muscle myogenesis.

The UV excision repair protein RAD23 homolog A isoform X1 (1.624, predicted) was found increased in aged rats compared to AGω3. RAD23 A is involved in protein turnover, DNA repair, and regulation of transcription [47]. We interpret the increased expression of RAD23 A in aged rats as a possible response to the accumulation of DNA damage to maintain cellular homeostasis. In support of this, aged rats may be dealing with more severe intracellular damage, many proteins connected to the proteolytic machinery were found up-regulated such as the proteasome (prosome, macropain) 26S subunit, non-ATPase, 9, isoform CRA_a (1.782); F-box only protein 3 (1.719); armadillo repeat-containing protein 8 (1.545). Additional evidence that the control group might be experiencing more damage than the AGω3 group can be found in the presence of proteins connected with an inflammatory status such as sialoadhesin isoform X1 (1.838, predicted); platelet glycoprotein Ib beta chain precursor (1.725); thrombospondin-1 precursor (1.706) and coagulation factor XII (1.502) (regulation of fibrinolysis, enrichment FDR 1.8E-02, FE 94.3).

At the level of the parenchyma, a plethora of proteins engaged in muscle function and contractility (sarcomere organization, enrichment FDR 1.3E-03, FE 42.8; myofibril assembly and striated muscle cell development, enrichment FDRs 3.5E-03, FEs 28.6 and 28.1) were found up-regulated in the AGCTL/AGω3 comparison, for example: myopalladin isoform X1 (2.294, predicted); alpha-actinin-3 (1.722); alpha-actinin-2 (1.682); actin-binding LIM protein 2 isoform X5 (1.676, predicted); myosin, heavy polypeptide 1, skeletal muscle, adult (1.632); actin-related protein 2/3 complex subunit 1B (1.548); and twinfilin-2 (1.745). The up-regulation of this network may be seen as an attempt to counteract age-related myofibrillar deterioration. Of note, also myosin-6 (1.674) showed increased abundance. The actin-dependent motor myosin-6 is *sui generis* among the different myosin variants performing a unique movement towards the minus pointed end of actin filaments [48]. Interestingly, even greater myosin-6 has been observed in solei muscles subjected to denervation, suggesting a possible correlation of myosin-6 and atrophic muscles [49]. In our data, the increased abundance of myosin-6 in control rats relative to AGω3 could suggest a protective and beneficial role of ω-3 PUFAs against muscle fiber damaging during aging.

In the AGCTL group several proteins pertaining to transcription, mRNA processing (spliceosome, enrichment FDR 7.4E-04, FE 18.1) and translation pathways were found up-regulated; however, Aω3 rats also show an increased expression of proteins participating in splicing events (spliceosome, enrichment FDR 3.0E-03).

Aged rats with ω-3 PUFAs increased by almost 2-fold the expression of similar to myocyte enhancer factor 2C, isoform CRA_d, partial (0.563, Mef2a), transcription regulatory factor whose relative abundance in AGω3 rats leads to an interesting reflection on whether ω-3 PUFAs might elicit a role in controlling satellite cells and inducing myogenesis in aged rats.

## Discussion

Hitherto, there is no “solo-treatment” that can prevent, delay, or reverse the damaging effects of aging on skeletal muscles. The modern eclectic approach aims at promoting musculoskeletal healthy aging from different angles by combining multiple interventions. Therefore, there is an urgent need to find novel molecular biomarkers of aging on skeletal muscle to better understand the etiology of this ineluctable phenomenon.

In this complex scenario, our study determined the impact of ω-3 PUFAs on the proteome of the soleus skeletal muscle during aging. Notably, ω-3 PUFAs have already emerged as worthwhile in other health states such as heart diseases; brain development; and inflammation [3].

The current bottom-up, global, shot-gun proteomic study investigated both the aging process and the response to ω3-PUFAs. Four different comparisons were set: ADCTL/ ADω3; AGCTL/AGω3; ADω3/AGω3; and ADCTL/AGCTL. These four comparisons showed remarkably different protein expressions as a result of diet and aging. Only proteins showing a differential abundance equal to or greater than 1.5-fold and a p ≤ 0.05 were considered for this analysis.

Aging impacts a plethora of different protein networks within skeletal muscle spanning bioenergetic, lipid metabolism, proteostasis, mRNA processing, sarcomeric structural proteins, and ECM. Our findings demonstrate that adult rats may be more energetically active than aged rats, with greater relative use of OXPHOS. While aerobic OXPHOS is the most effective pathway to obtain energy, it also generates reactive byproducts which may be difficult to manage for aged rats. Indeed, aged rats show an increased expression of structural proteins belonging to intermediate filaments and sustained protein synthesis, which may be an attempt to maintain general proteostasis. Therefore, processes pertaining to mRNA processing and translation were also increased during aging. Lastly, at the level of the ECM, different collagen molecules showed an increased abundance during aging, inferring a potential effect of senescence not only confined to the muscular parenchyma, but also to the stromata.

Similar trends in proteome changes were found between the ADCTL/AGCTL and ADω3/AGω3 comparisons. However, in the latter comparison AGω3 rats express a greater presence of proteins involved in chromatin-related phenomena, such as methylation or histone deacetylation, introducing scenarios on nutri-epigenomics and epigenetic aging. Differently, ADω3 rats may invest the antioxidant properties of ω-3 PUFAs to support mitochondrial integrity and homeostasis (accompanied by an increase of mTOR), undergoing a metabolic rewiring which leans towards an aerobic, respiratory phenotype.

Regarding the diet groups, the effects induced by ω-3 supplementation seem to be greater in aged rats than in the adult. As expected, the modest changes in the adult group can be interpreted as a lesser necessity for the beneficial properties of ω-3 PUFAs.

In contrast, aged rats greatly benefit from the effects of ω-3 PUFAs. Evidence can be found in the different proteomic abundances of AGCTL and AGω3 rats. The control group shows an accumulation of DNA damage repair along with increased protein synthesis and degradation. Even at the sarcomeric level, a plethora of different structural proteins are up-regulated, most likely as a response to the myofibrillar deterioration due to aging. Conversely, aged rats supplemented with ω-3 PUFAs do not present this damage to the same extent as the control group. The almost 2-fold differential expression of similar to myocyte enhancer factor 2C, isoform CRA_d, partial (0.563, Mef2a) is also of interest. With Mef2a being more expressed in the AGω3 group, interesting scenarios regarding a potential role of ω-3 PUFAs in controlling muscle satellite cells arise. In support of our finding that may link ω-3 PUFAs with skeletal muscle myogenesis during aging through Mef2a, review articles have proposed additional hypothesis on how ω-3 PUFAs could influence muscle growth and regeneration [50,51].

Taken together, our data suggest a potential beneficial and protective effect of ω-3 PUFAs on the proteome during aging. Being that our study was conducted on male rats, it would be worthwhile investigating if a comparable response is also evoked in female rats. There may be a gender-muscle-specific response to ω-3 PUFAs. Furthermore, collecting more timepoints, in addition to 7–8- and 22-month-old rats, could be instrumental for estimating at which stage of life ω-3 PUFAs commence exerting their beneficial effects. This, combined with existing recommendations such as exercise and dietary restriction, could ameliorate the symptoms of aging and improve the quality of life. In this scenario, a non-invasive dietary intervention with limited side-effects could make the difference in achieving successful and healthy aging.

## Supporting information

S1 Table Soleus mass comparisons and sarcopenia evaluation. The tables show a lack of sarcopenia (age-related loss of mass). **(A)** age X diet ANOVA indicating that no significant effects or interactions for age or diet on soleus mass were observed; **(B)** means and SE, units (mg), y (FO), b (control diet); **(C)** age X diet ANOVA indicating the no significant effects or interactions on the “sarcopenia index” (SI) [11] were observed; **(D)** means and SE, “sarcopenia index” (SI) [11], units (mg/g, body mass), y (FO), b (control diet).
(PDF)

S1 Fig Immunoblots detecting the expression of NADH dehydrogenase ubiquinone flavoprotein 2. Means with standard deviations (SD) are reported. Groups and arbitrary units (pixel intensities) are reported on the x and y axes, respectively.
(PDF)

S2 Fig Immunoblots detecting the expression of histone H3. Means with standard deviations (SD) are reported. Groups and arbitrary units (pixel intensities) are reported on the x and y axes, respectively.
(PDF)

S3 Fig Immunoblots detecting the expression of GAPDH. Means with standard deviations (SD) are reported. Groups and arbitrary units (pixel intensities) are reported on the x and y axes, respectively. As expected GAPDH was predominantly expressed in the supernate (sarcoplasmic) fraction. In line with the LC-MS/MS data, GAPDH did not show both significant (p ≤ 0.05) and differential (+/-1.5-FC) abundances between the comparisons.
(PDF)

S4 Fig Reversible MemCode stain of the PVDF membranes. Each fraction of the total proteome transferred onto the PVDF membranes is normalized to total protein loading determined via reversible stain. Each membrane shows one of the 4 comparisons, with each comparison containing 2 conditions (five biological replicates per group). Lanes 2 and 8 are controls and lane 1 is MW marker (kDa).
(TIF)

S1 File List of both significant and differential abundance proteins in ADCTL-AGCTL supernate. (XLSX)

S2 File List of both significant and differential abundance proteins in ADCTL-AGCTL pellet. (XLSX)

S3 File List of both significant and differential abundance proteins in ADω3/AGω3 supernate. (XLSX)

S4 File List of both significant and differential abundance proteins in ADω3/AGω3 pellet. (XLSX)

S5 File List of both significant and differential abundance proteins in ADCTL/ADω3 supernate. (XLSX)

S6 File List of both significant and differential abundance proteins in ADCTL/ADω3 pellet. (XLSX)

S7 File List of both significant and differential abundance proteins in AGCTL/AGω3 supernate. (XLSX)

S8 File List of both significant and differential abundance proteins in AGCTL/AGω3 pellet. (XLSX)

S9 File Raw images. (PDF)

## Abbreviations

ADCTL: adult control
ADω3: adult ω-3
AGCTL: aged controls
AGω3: aged ω-3
CAC: citric acid cycle
CTL: control
DHA: docosahexaenoic acid
ECM: extracellular matrix
EPA: eicosapentaenoic acid
FA: fatty acids
FC: fold change
FDR: false discovery rate
FE: fold enrichment
FO: fish oil
OXPHOS: oxidative phosphorylation
PTM: post-translational modification
ROS: reactive oxygen species
RT: room temperature
SD: standard deviation
SDS-PAGE: sodium dodecyl sulphate-polyacrylamide gel electrophoresis
TMT: tandem mass tag
ω-3 PUFAs: omega-3 polyunsaturated fatty acids
