# Supplementary material for: Proteomes of aging and omega-3 supplementation in rat soleus skeletal muscle
Source: PLoS One. 2025 May 27;20(5):e0323602. doi: 10.1371/journal.pone.0323602 (PMC12111612; doi:10.1371/journal.pone.0323602)
Supplement: S1 Table — The tables show a lack of sarcopenia (age-related loss of mass). (A) age X diet ANOVA indicating that no significant effects or interactions for age or diet on soleus mass were observed; (B) means and SE, units (mg), y (FO), b (control diet); (C) age X diet ANOVA indicating the no significant effects or interactions on the “sarcopenia index” (SI) [11] were observed; (D) means and SE, “sarcopenia index” (SI) [11], units (mg/g, body mass), y (FO), b (control diet). (PDF) [file pone.0323602.s001.pdf]

(A)

Tests of Between-Subjects Effects

Dependent Variable: SolMass

Source	Type III Sum of Squares	df	Mean Square	F	Sig.
Corrected Model	2330.683[a]	3	776.894	.844	.487
Intercept	692646.649	1	692646.649	752.225	<.001
Diet	7.804	1	7.804	.008	.928
Age	259.740	1	259.740	.282	.601
Diet * Age	1666.955	1	1666.955	1.810	.194
Error	17495.143	19	920.797		
Total	806261.000	23			
Corrected Total	19825.826	22			

a. R Squared = .118 (Adjusted R Squared = -.022)

(B)

Estimates

Dependent Variable: SolMass

Diet	Age	Mean	Std. Error	95% Confidence Interval	
				Lower Bound	Upper Bound
b	Adult	189.286	11.469	165.280	213.291
	Aged	178.333	17.519	141.665	215.002
y	Adult	172.429	11.469	148.423	196.434
	Aged	197.667	12.388	171.738	223.595

(C)

Tests of Between-Subjects Effects

Dependent Variable: SI

Source	Type III Sum of Squares	df	Mean Square	F	Sig.
Corrected Model	.007[a]	3	.002	.976	.425
Intercept	2.358	1	2.358	972.028	<.001
Diet	.000	1	.000	.056	.815
Age	.000	1	.000	.104	.751
Diet * Age	.006	1	.006	2.440	.135
Error	.046	19	.002		
Total	2.758	23			
Corrected Total	.053	22			

a. R Squared = .133 (Adjusted R Squared = -.003)

(D)

Estimates

Dependent Variable: SI

Diet	Age	Mean	Std. Error	95% Confidence Interval	
				Lower Bound	Upper Bound
b	Adult	.363	.019	.324	.402
	Aged	.322	.028	.263	.382
y	Adult	.324	.019	.285	.363
	Aged	.351	.020	.309	.393
