# Supplementary material for: Proteomes of aging and omega-3 supplementation in rat soleus skeletal muscle
Source: PLoS One. 2025 May 27;20(5):e0323602. doi: 10.1371/journal.pone.0323602 (PMC12111612; doi:10.1371/journal.pone.0323602)
Supplement: S1 Fig — Means with standard deviations (SD) are reported. Groups and arbitrary units (pixel intensities) are reported on the x and y axes, respectively. (PDF) [file pone.0323602.s002.pdf]

NADH ADCTL-AGCTL SUP

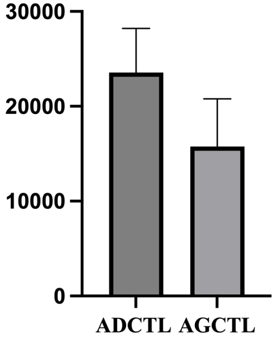

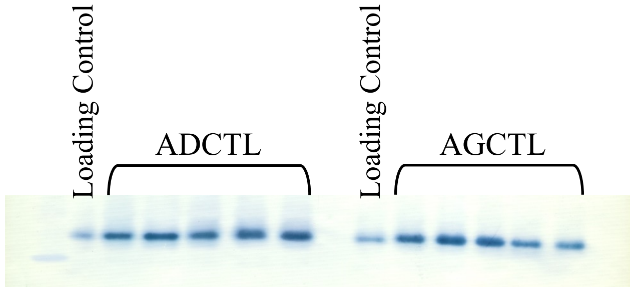

p-value (*): 0.0346

NADH ADω3-AGω3 SUP

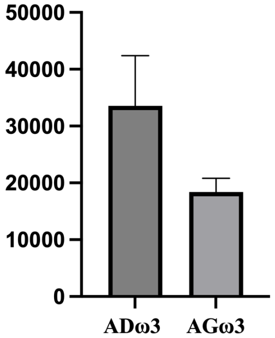

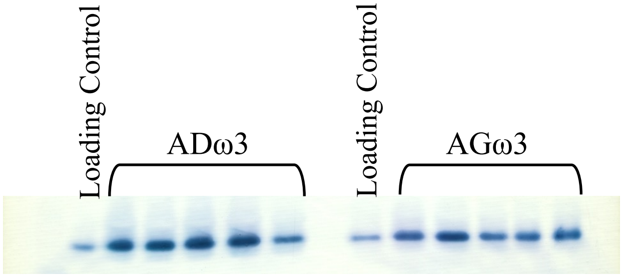

p-value (*): 0.0160

NADH ADCTL-AGCTL PEL

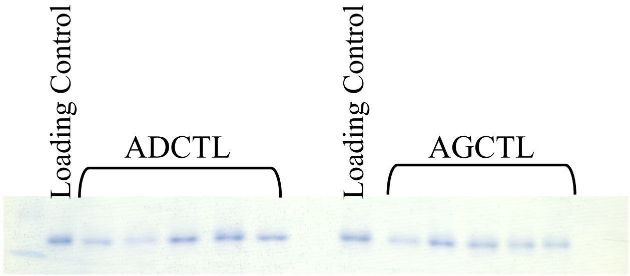

Below LOD

NADH ADω3 -AGω3 PEL

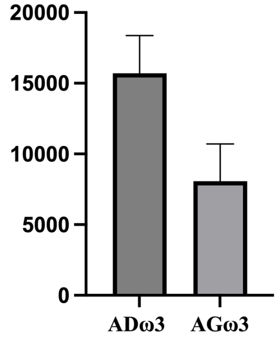

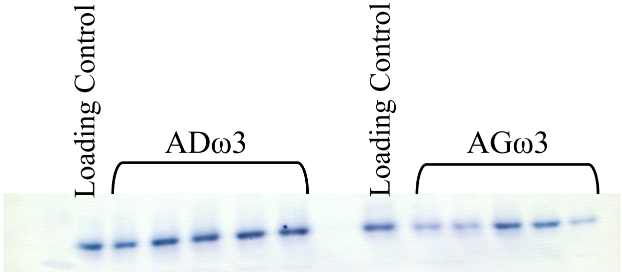

p-value (**): 0.0018
