# Supplementary material for: Proteomes of aging and omega-3 supplementation in rat soleus skeletal muscle
Source: PLoS One. 2025 May 27;20(5):e0323602. doi: 10.1371/journal.pone.0323602 (PMC12111612; doi:10.1371/journal.pone.0323602)
Supplement: S2 Fig — Means with standard deviations (SD) are reported. Groups and arbitrary units (pixel intensities) are reported on the x and y axes, respectively. (PDF) [file pone.0323602.s003.pdf]

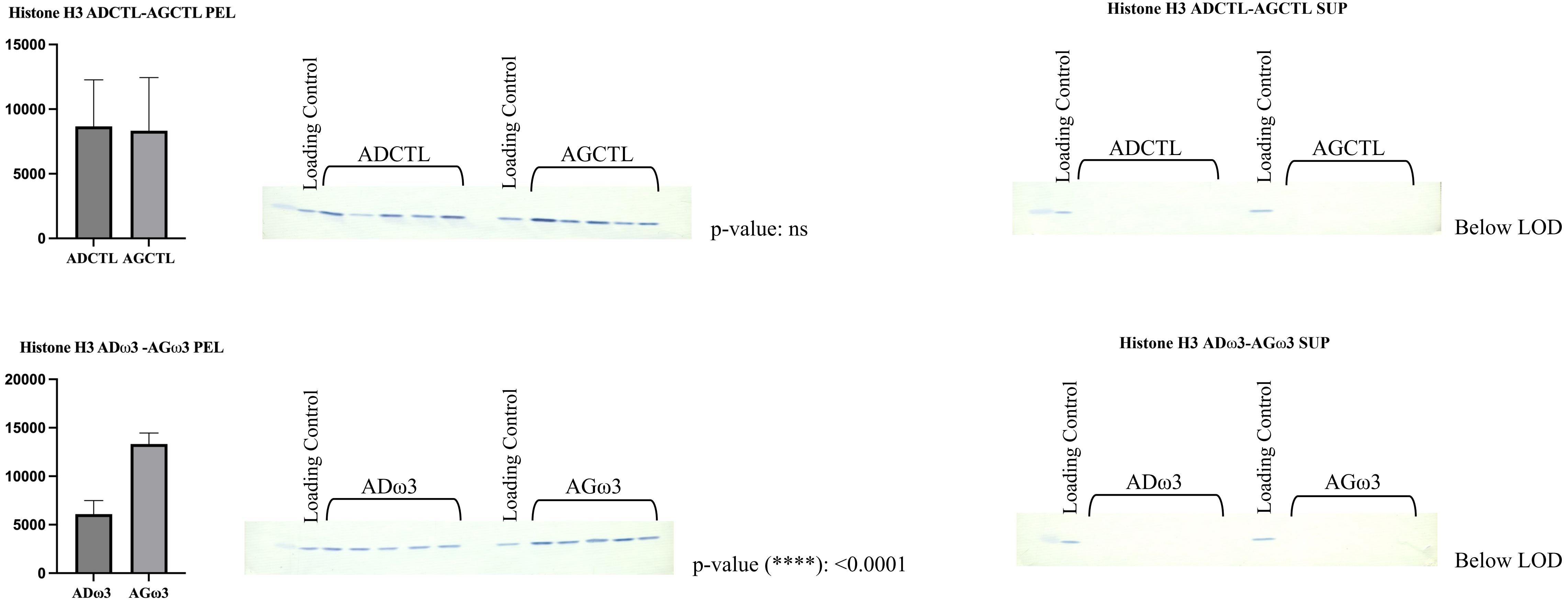
Histone H3 ADCTL-AGCTL PEL
15000
10000
5000
0
ADCTL AGCTL
Loading Control
ADCTL
Loading Control
AGCTL
p-value: ns
Histone H3 ADCTL-AGCTL SUP
Loading Control
ADCTL
Loading Control
AGCTL
Below LOD
Histone H3 ADω3 -AGω3 PEL
20000
15000
10000
5000
0
ADω3 AGω3
Loading Control
ADω3
Loading Control
AGω3
p-value (****): <0.0001
Histone H3 ADω3-AGω3 SUP
Loading Control
ADω3
Loading Control
AGω3
Below LOD
