# Supplementary material for: Proteomes of aging and omega-3 supplementation in rat soleus skeletal muscle
Source: PLoS One. 2025 May 27;20(5):e0323602. doi: 10.1371/journal.pone.0323602 (PMC12111612; doi:10.1371/journal.pone.0323602)
Supplement: S3 Fig — Means with standard deviations (SD) are reported. Groups and arbitrary units (pixel intensities) are reported on the x and y axes, respectively. As expected GAPDH was predominantly expressed in the supernate (sarcoplasmic) fraction. In line with the LC-MS/MS data, GAPDH did not show both significant (p ≤ 0.05) and differential (+/-1.5-FC) abundances between the comparisons. (PDF) [file pone.0323602.s004.pdf]

GAPDH ADCTL-AGCTL SUP

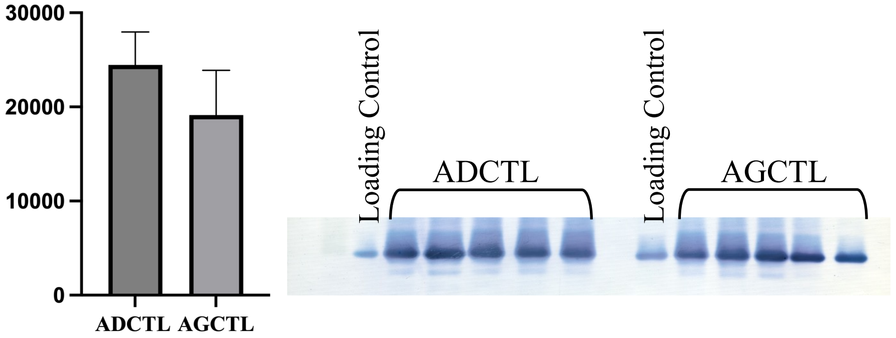

p-value: ns

GAPDH ADω3-AGω3 SUP

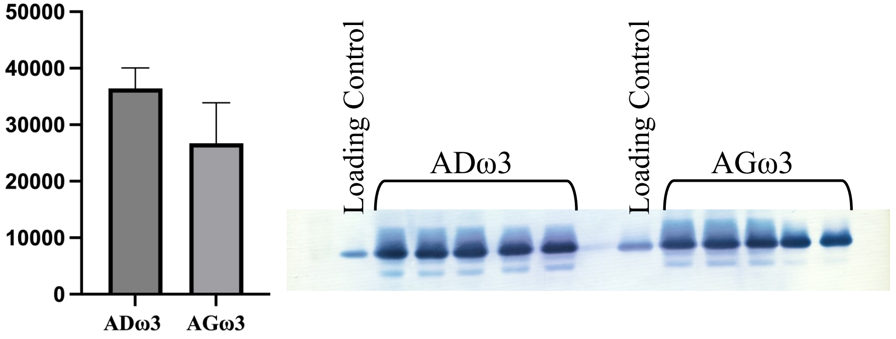

p-value (*): 0.0361

GAPDH ADω3 -AGω3 PEL

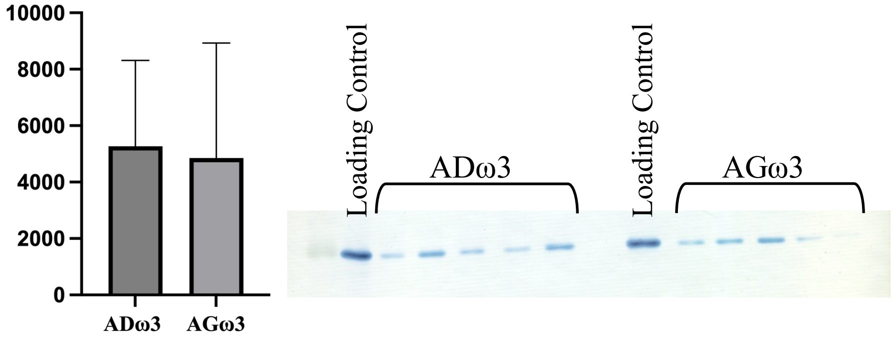

p-value: ns

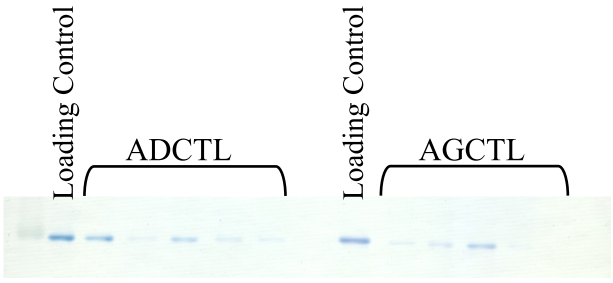

GAPDH ADCTL-AGCTL PEL

Below LOD
