# Supplementary figures and images for: Proteomes of aging and omega-3 supplementation in rat soleus skeletal muscle
Source: PLoS One. 2025 May 27;20(5):e0323602. doi: 10.1371/journal.pone.0323602 (PMC12111612; doi:10.1371/journal.pone.0323602)

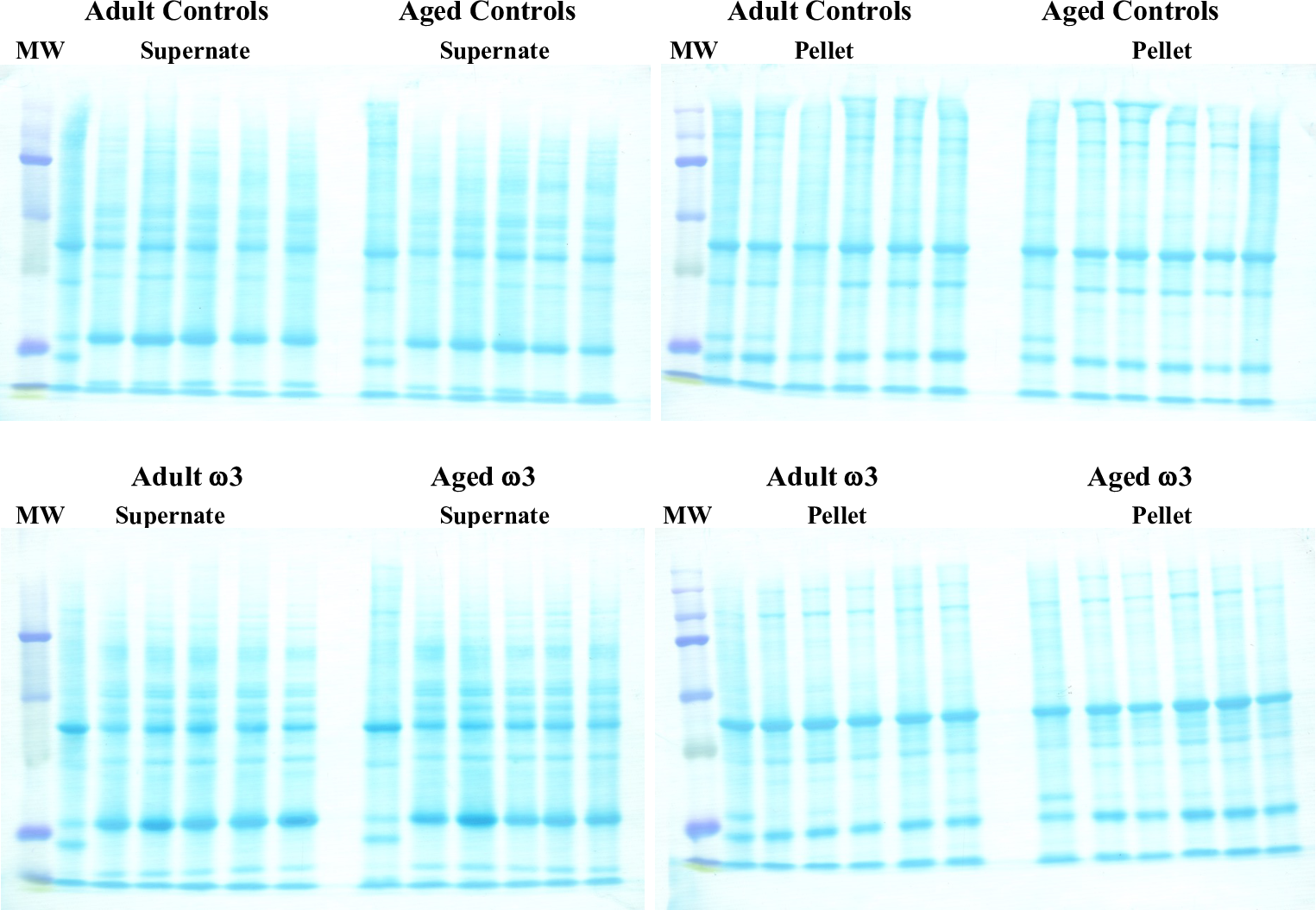

Supplement: S4 Fig — Each fraction of the total proteome transferred onto the PVDF membranes is normalized to total protein loading determined via reversible stain. Each membrane shows one of the 4 comparisons, with each comparison containing 2 conditions (five biological replicates per group). Lanes 2 and 8 are controls and lane 1 is MW marker (kDa). (TIF) [file pone.0323602.s005.tif]
