# Supplementary material for: Proteomes of aging and omega-3 supplementation in rat soleus skeletal muscle
Source: PLoS One. 2025 May 27;20(5):e0323602. doi: 10.1371/journal.pone.0323602 (PMC12111612; doi:10.1371/journal.pone.0323602)
Supplement: S9 File — (PDF) [file pone.0323602.s014.pdf]

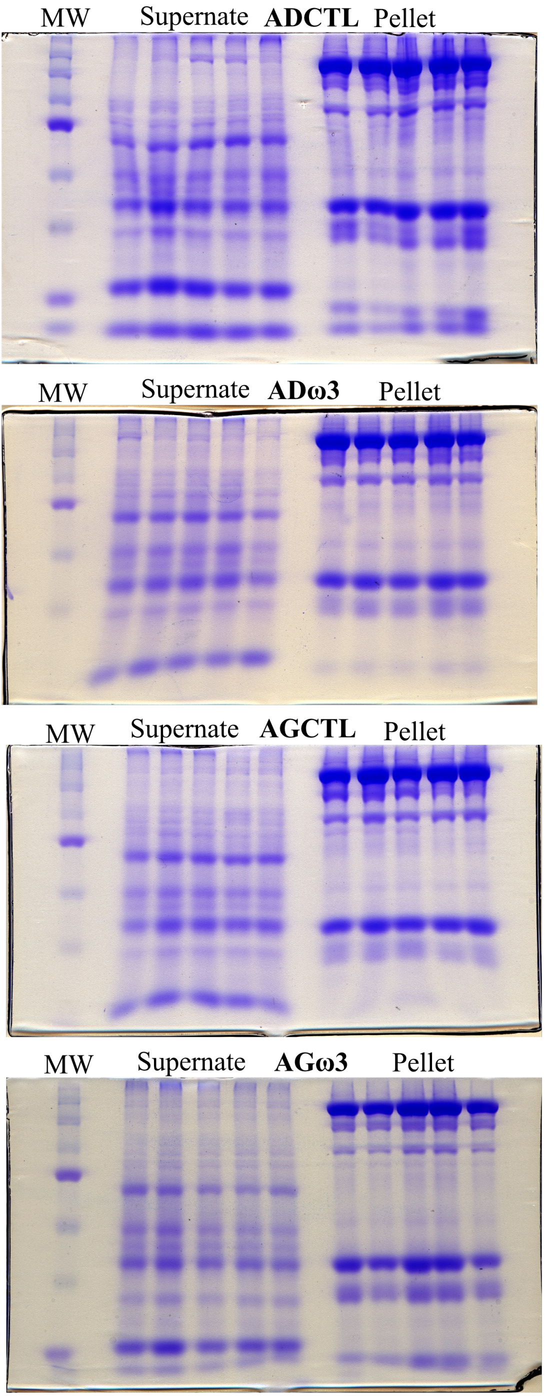

Fig 1 was generated from these original gel images

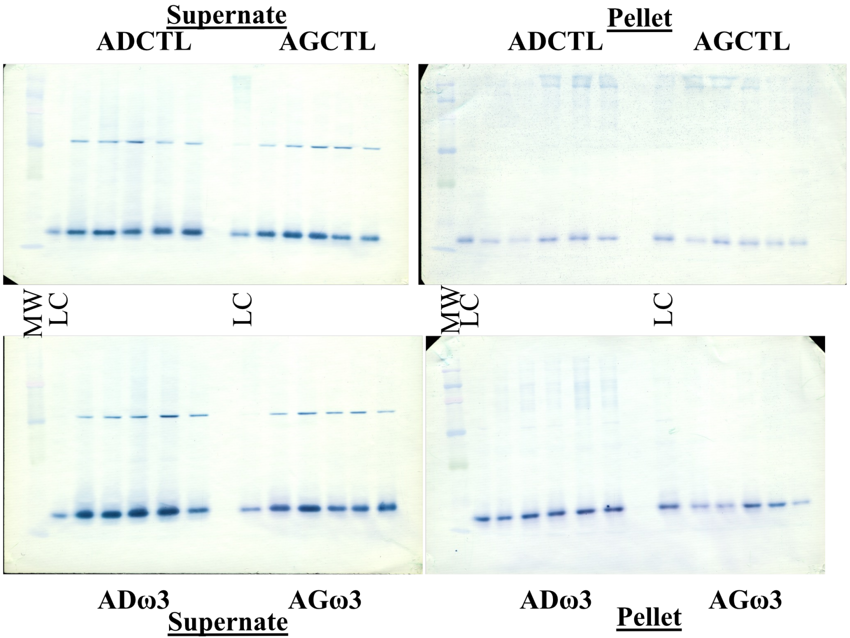

Immunoblots of NADH dehydrogenase ubiquinone flavoprotein 2. Fig 4 and S1 Fig were adapted from these original blots

LC= Loading Control

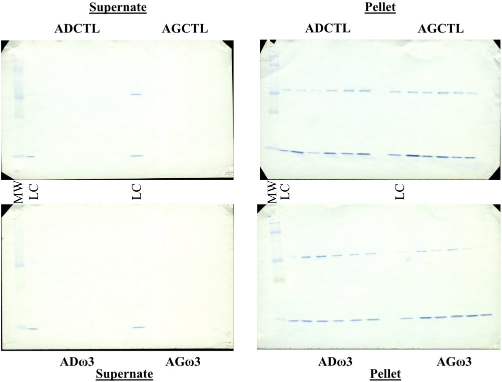

Immunoblots of Histone H3. Fig 6 and S2 Fig were adapted from these original blots.

LC= Loading Control

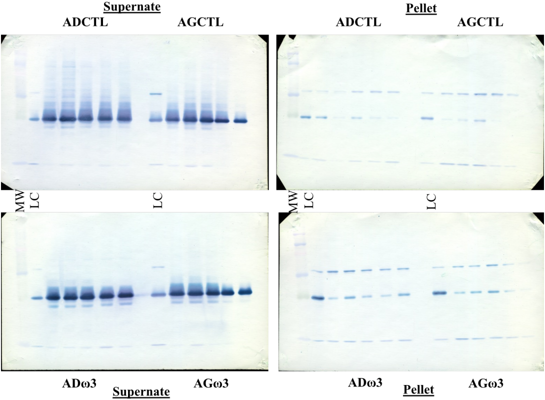

Immunoblots of GAPDH. S3 Fig was adapted from these original blots.
LC= Loading Control

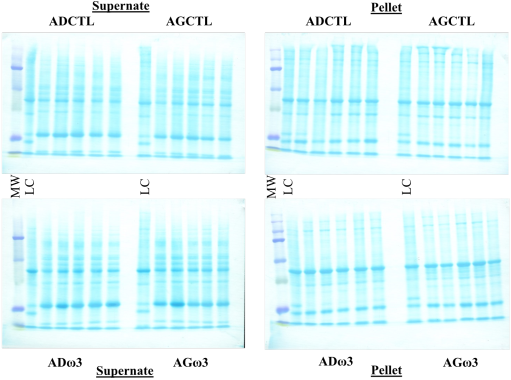

Reversible MemCode stain of the blots. S4 Fig was generated from these original blots.

LC= Loading Control
